# Deep-Stacking Network Approach by Multisource Data Mining for Hazardous Risk Identification in IoT-Based Intelligent Food Management Systems

**DOI:** 10.1155/2021/1194565

**Published:** 2021-11-10

**Authors:** Jianlei Kong, Chengcai Yang, Jianli Wang, Xiaoyi Wang, Min Zuo, Xuebo Jin, Sen Lin

**Affiliations:** ^1^School of Artificial Intelligence, Beijing Technology and Business University, Beijing 100048, China; ^2^National Engineering Laboratory for Agri-Product Quality Traceability, Beijing 100048, China; ^3^Intelligent Equipment Research Center, Beijing Academy of Agriculture and Forestry Sciences, Beijing 100097, China

## Abstract

Food quality and safety issues occurred frequently in recent years, which have attracted more and more attention of social and international organizations. Considering the increased quality risk in the food supply chain, many researchers have applied various information technologies to develop real-time risk identification and traceability systems (RITSs) for preferable food safety guarantee. This paper presents an innovative approach by utilizing the deep-stacking network method for hazardous risk identification, which relies on massive multisource data monitored by the Internet of Things timely in the whole food supply chain. The aim of the proposed method is to help managers and operators in food enterprises to find accurate risk levels of food security in advance and to provide regulatory authorities and consumers with potential rules for better decision-making, thereby maintaining the safety and sustainability of food product supply. The verification experiments show that the proposed method has the best performance in terms of prediction accuracy up to 97.62%, meanwhile achieves the appropriate model parameters only up to 211.26 megabytes. Moreover, the case analysis is implemented to illustrate the outperforming performance of the proposed method in risk level identification. It can effectively enhance the RITS ability for assuring food supply chain security and attaining multiple cooperation between regulators, enterprises, and consumers.

## 1. Introduction

With the global intention of the food security concept shifted from fighting hunger and malnutrition to healthy living and food, the food supply chain is not only an important issue of public health and trade but also one of the most challenging sustainability issues, which significantly affects the stable development of national sovereignty, economy, and society [1].

Nowadays, the modern food supply chains usually contain complicated transport links including planting, harvesting, handling, storage, transport, loading, packaging, and marketing, over an extended distance and different partners from producer to consumer as a result of global food trade and economy. Ensuring the quality and security of food products in the supply chain systems is the most likely and effective approach to solve many problems such as covering climate change, biodiversity loss, land degradation, and pollution [2]. For example, the soybean supply chain is potentially responsible for global anthropogenic carbon emissions due to miscellaneous ecological and social impacts, ranging from uncontrolled deforestation, biodiversity consumption, and inefficient exploitation of natural resources to human and livestock's food waste. Therefore, it is obvious that the safe and sustainable development of the food supply chain is nowadays playing a vital role in human health, steady economic development, and environmental protection [3].

Nevertheless, after a series of foodborne disease outbreaks and food safety incidents occurring in the past few decades, the food supply chain has faced increased quality and safety risks. With extended haul distance and long-term storage time from the planter to the consumer across different stages and industries, various chemical and microbial contaminations, as well as foodborne viruses, have easily invaded each supply stage of the food supply chain, which have severely threatened the food safety and disrupted the stable sustainability of food supply [4]. In particular, excessive enterprises and companies are now joining the food supply chain which results in that the food products should go through a number of interested parties before reaching the final consumers.

In such a complicated and invisible supply chain, many potential factors including temperature, humidity, pasteurization, or heat treatments have increased the unpredictable risks in the food supply chain since not all participants can strictly ensure their process operations and management conform to all the standards. As a result, the unsafe vulnerabilities of food supply chains have been ulteriorly aggravated due to the inherent multitiered cascading effect [5]. Therefore, ensuring food security and quality along the supply chain has become an important and substantiated approach to satisfy the uptrending demand of customers for high-quality food with safety guarantees and ecological sustainability.

With the development of information and intelligence technologies such as the Internet of Things (IoT), automated robots and equipment, cloud computing, and smart factory management systems, the modern food supply chains are struggling to make the most of innovative technologies to increase the interconnected efficiency of organizations and support sustainable practices in the form of products and services through various food supply linkages [6]. As one of the important roles, the risk identification and traceability system (RITS) along the food supply chain on the basis of IoT and related technologies that aim at monitoring, sharing, and connecting anything, anytime, and anywhere has become a popular recognized approach for food safety and quality assurance since it can monitor various foodborne hazards on time, identify potential food safety issues, and assess food contamination status by providing abundant sensor data and surveillance information [7].

At the same time, the system is also a valuable tool in supporting sustainable development in the food supply chain from producers to consumers, which can efficiently reduce resource waste, replace extensive operations with carbon concentration, and eventually avoid food safety incidents to mitigate climate change and other negative environmental and social impacts of the whole process [8]. The above benefits associated with lower-cost distribution, reducing recall, and inventory expenses and averting many hidden troubles that possibly existed have made most enterprises to broadly implement the RITS technology in their food supply chain.

Therefore, by taking advantage of deep learning techniques, the present study proposed the novel deep-stacking network (DSN) to establish an improved identification and traceability system, which aims to promote reliable food safety and sustainable assurance of the whole supply chain. This method could not only automatically process a large amount of monitoring biosignals collected by the multisource IoT sensors and instruments to evaluate food quality but also mine the interaction of multiple hazards and related risk factors to derive preferable risk grades of food supply chains.

This paper is organized as follows: in Section 2, this paper introduces the work related to food safety risk prediction. Then, Section 3 outlines the overall method of the proposed deep-stacking network and IoT-based food system. In Section 4, the comparative risk recognition results of different hazards are presented, and the risk distribution analysis in terms of grain supply chains is discussed. Finally, we conclude the paper with the future implementation prospects in Section 5.

## 2. Related Works

### 2.1. Food Safety Traceability Frameworks and Construction Standards

Nowadays, there are several food safety traceability frameworks and construction standards, e.g., the Rapid Alert System for Food and Feed (RASFF) system approved by the European Union, Food Safety System Certification (FSSC) framework, and the GEMS/Food Contamination Monitoring and Assessment Programme (GEMS/Food) established by the World Health Organization (WHO), which aim to effectively manage accident safety risks, promote quality improvement, maximize consumer trust, support market efficiency, and achieve precision agriculture within the supply chain [9]. Similarly, China has rapidly developed a fully functional national surveillance system monitoring various hazards based on the Hazard Analysis Critical Control Point (HACCP) standard [10]. The system currently provides traceability guarantee with early-warning analysis for chemical and microbiological hazards, foodborne diseases, and other potential risk factors, further preventing large-scale food safety events and safeguarding people's lives and health.

Although lots of shared information have strengthened the communication and cooperation between each node of the food supply chain, these systems are not enough to handle the increasing challenges of current food supply chains, such as flexibility, compatibility, credibility, and comprehensive decision-making [11]. On the one hand, the growing information collected by various sensors of the IoT or shared by different supply chain partners enables factories to real-time solve the traceability problems of the food supply chain and improve food quality and safety, but many managers and researchers have now figured out that the plentiful data sources stored in the system are not used effectively since the existing RITS lacks an effective unified method to timely process such large-scaled, multisourced, and heterogeneous data of the whole supply chain, which are very valuable in today's highly competitive market [12].

Making full use of these data will support members' operating abilities in hazard analysis, enhance enterprise competitiveness in risk prewarning, and promote the effectiveness of food safety supervision and management [13]. Therefore, a complete RITS must contain a standard evaluation and prediction model that automatically handles huge monitored data collected from the IoT network and diagnoses the possible risky problem, which affects the performance of the whole food supply chain by reducing inventory costs as well as the bullwhip effect across the long distances and changing environment.

On the other hand, most researchers have performed risk identification and prediction of the food supply chain from a local perspective, which means each supply chain partner in the food system can successfully find the quality and safety problems in their own production process [14]. However, existing methods still lack the comprehensive capacity guaranteeing the safety and sustainability in the food industry since different component modes of the food supply chain are scarce and incomplete [15]. Normally, an unsafe product will cross a number of interested parties and multitiered links to reach the end user along the food supply chain.

Many foodborne mycotoxins contaminated in those unsafe food products may spread in a fast-growing number under certain circumstances since the food supply chain naturally had the migration structure and dynamic cascading function. A classical static-method-based RITS failed to anticipate a wide variety of risk factors influencing food safety and quality throughout the whole supply chain operations and members [16]. Thus, the RITS assisted by intelligent IoT technology might already have been used in operational management processes, but an effective approach to identify and prewarn risk grades of various factors under the macroperspective is still absent in the whole food supply chain.

### 2.2. Risk Prediction Based on Machine Learning

In order to strengthening the safety of the extended food supply chain, a variety of new approaches have been introduced to meet these challenges. Machine learning models including the random forest (RF), support vector machine (SVM), decision tree (DT), *k*-nearest neighbour (KNN), and artificial neural network (ANN) [17–19] are applied to generalize the main food supply stages and processes with the implementation of the IoT paradigm, which generally explores the risky criticalities of each supply chain. Then, the connections between the nearby supply chain entities are established to assess the risk profile of the RITS as a whole, instead of focusing on some specific supply links. For example, RF and logistic regression (LR) were used to rank important variables offered by experts to classify the persistence of *Listeria monocytogenes* in six retail delicatessens [20].

Similarly, SVM-based methods have been explored to access risk levels for making sure the supply safety of dairy production in northeastern Brazil [21]. Furthermore, many studies have tried to apply multistacked algorithms including DT, KNN, and ANN to rank the risk levels of chemical hazards present in food products, which all achieved better results compared to statistical methods or manual analysis [22, 23].

Although classical machine learning methods would offer an effective approach for food safety supervision to deal with perishable products and unpredictable supply variations to some extent, the intelligence degree of existing food systems is still too far away from meeting stringent food safety requirements, as well as assisting managers in making correct decisions dynamically for identification and mitigation of global risks [24]. Especially, since most companies now are a part of the food supply chain, perishable food products usually cross a number of interesting links before reaching the last consumers. Traditional methods used in the RITS make such systems fail to control the quality of food products since enormous sensor data are not effectively utilized to analyze the interactions between hazards joining the supply chains [25, 26].

### 2.3. Risk Prediction Based on Deep Learning

In recent years, as RITS management has been rapidly developed toward Industry 4.0 on the basis of artificial intelligence and IoT technologies, deep learning methods including AlexNet [27], GoogLeNet [28], VGG [29], and ResNet [30] are playing a vital role in this transition. The core concept of deep learning technology is stacking multilayered neural networks with various training tricks (including data enhancements, flexible structural designs, complex loss functions, and parameter optimization strategies), which have been demonstrated to be effective for handling food risk identification, assessment, and traceability problems on the basis of abundant data stored in the RITS [31–33].

Meanwhile, some researchers have pointed out that deep learning methods are very preferable to make full use of these valuable data to enhance enterprise competitiveness, as well as gain the trust of consumers in highly competitive markets [34, 35]. However, deep learning methods combined with IoT information analysis are yet to assess potential risks of hazardous substances in the whole supply chain [36, 37]. Therefore, it is necessary for the RITS to conduct a risk assessment model from a macroperspective, which could comprehensively utilize and analyze large amounts of sensor data to estimate the hazardous levels of different contaminants and to ensure the safety and quality of food supply chains [38, 39].

Therefore, by taking advantage of deep learning techniques, the present study proposed the novel deep-stacking network (DSN) to establish an improved identification and traceability system, which aims to promote reliable food safety and sustainable assurance of the whole supply chain. This method could not only automatically process a large amount of monitoring biosignals collected by the multisource IoT sensors and instruments to evaluate food quality but also mine the interaction of multiple hazards and related risk factors to derive preferable risk grades of food supply chains. With the comprehensive evaluation of food quality, the food system could manage supply chain processes and participants effectively to avoid possible quality accidents and reduce regulatory costs. Meanwhile, the customers also obtain a rare glimpse from the inside of a hierarchical supply chain as well as the basis of a sharable and scientific risk analysis, which improve their confidence in making intelligent purchasing decisions and buying high-quality food products to meet a healthy daily diet.

## 3. Materials and Methods

Food safety inspection is a huge and complex Internet of Things system with various sensors. In this paper, all kinds of sensor data are processed scientifically. On this basis, we propose the deep-stacking network to forecast and estimate the food safety risks. This paper achieves the proposed method with three stages. Firstly, a multigranularity padded-scanning method is adopted to generate input vectors with adjustable dimensions in the first stage, and the *K*-fold cross-validation method is applied to expand the utilization of limited training data in the second stage. Finally, the deep-stacking network is consolidated to extract a list of food safety prewarning features, which speed up the mining process and improve identification performance in the present study.

### 3.1. Risk Identification and Traceability System

**Figure 1:**
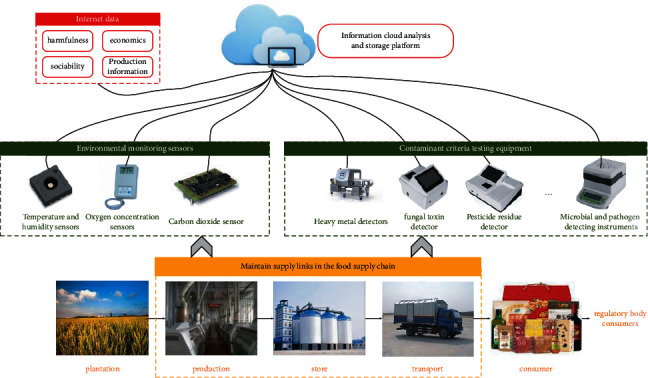
IoT-based risk identification and traceability system for the grain supply chain.

As the response speed of the food system is very important, we proposed an improved risk identification and traceability based on IoT and nephanalysis technologies, which can take advantage of information sharing to coordinate the operation among nodes of the supply chain, to ensure the food safety prewarning system operates efficiently and safely. This system collected a large amount of monitoring data from various links through various equipment, including temperature and humidity sensors, oxygen concentration sensors, and carbon dioxide concentration sensors, as well as different pollutant detection equipment (as shown in Figure 1). In the production, storing, and transportation chain, this research cooperated with the food enterprises to obtain various important environmental monitoring data, such as temperature, humidity, light exposure, oxygen, and carbon dioxide concentrations, as well as production information including type, weight, expiration date, manufacturer, and producing place, which were measured by a large number of multisource sensors in the grain processing factories.

Meanwhile, we applied various contaminant testing equipment to extract the key hazard data related to food safety including heavy metals, microorganisms, mycotoxins, and pesticide residues implicit in grain products at each supply stage of the grain supply chain. Among them, the heavy metal detector uses anodic stripping voltammetry to detect elements and contents of different heavy metals in grain products such as cadmium, mercury, arsenic, and aluminum, while the mycotoxin detector is used to rapidly detect aflatoxin-B1 (AFB1), ochratoxin A (OTA), zearalenone (ZON), deoxynivalenol (DON), T2 toxin, fumonisin, etc., in rice, wheat, corn, and other grains with high accuracy and sensitivity. The pesticide residue fast tester detects various organophosphorus and hydrogen-formatted pesticides in grain sales and consumption sites such as farmers' markets, supermarkets, planting bases, restaurants, and laboratories.

Additionally, the microbial and pathogen detecting instruments use immunoconcentration technology to carry out antibody capture, concentrated release, purification, separation, and automatic detection of pathogenic bacteria including coliforms (COLI), *Salmonella, Listeria*, methicillin-resistant *Staphylococcus aureus* (MRSA), and other bacteria in grain products. All data are transmitted to the information cloud analysis and storage platform through TCP/IP and wireless approaches, which is the center for data collection, information storage, and exchanging from all relevant steps in the food supply chain. The grain product types included rice, wheat flour, corn, roughage, and other grain-processed products, which cover the most important grain structure of Chinese consumers.

With the RITS operated in the period from March 2016 to August 2019, there are a lot of grain data covering 26 primary grain-producing areas recorded in the food system. Those provinces are the highest consumption areas because they are also the most densely populated. Due to the redundant and abnormal information recorded and collected through the IoT platform, we uniformly integrate different data from various devices and extract some important attributes to construct the retrospective database with regular formats and value ranges. The final database and detail attributes in uniformed styles are shown in Table 1.

Then, in order to get a reasonable hazard level, this paper consults the relevant literature, studies the combination of international standards and domestic standards, consults relevant experts in the field of food safety, and crawls public data on the internet. In order to obtain a more comprehensive, scientific, and credible risk assessment tool so proper conclusions can be made, We hope to enrich the real-time IoT datasets with information derived from hazardous, social, economic, and regulatory food safety aspects so that a retrospective database can cover more dimensional analyses and multiple participant perspectives.

Therefore, these qualitative indicators such as social attention, harm degree, and accessibility of supervision are covered. And quantitative indicators are also considered, for example, the province's total annual output, food production, and consumer prices. The expanded attributes of the final database are shown in Table 2. Based on the above situations, the risk grade of each product is divided into three areas (safety, warning, and danger) with eight levels: high-safety level (I), safety level (II), warning level (III), low-risk level (IV), medium-risk level (V), high-risk level (VI), higher risk level (VII), and highest risk level (VIII), where levels I and II belong to the safe area, level III belongs to the warning area, and others are the danger area.

### 3.2. Data Analysis and Preprocessing

#### 3.2.1. Unstructured Data Encoding

There are a lot of unstructured data recorded in the retrospective database, which were collected from different sources and stored in various formats. Moreover, with common operating errors caused by incorrect human interference, the database is filled with unauthentic and abnormal data unreflecting the real situations of grain quality security in the supply chain, which has just reduced the prewarning and traceable performance of the food system. It is necessary to add some data analyses and preprocessing to extract encoding rules and uniform multisource data format before proceeding with the sequential risk evaluation.

Abnormal and unstructured data cannot be directly input to the computer for calculation, so it is necessary to digitize such data through coding. In order to encode the abnormal and unstructured data, the dictionary vectorizer (DictV) and one-hot encoder (OneH) are combined to complete the encoding process so that we could input the correct data to the grain RITS as cases which are employed to mining risk association rules and prewarning information directly. The dictionary vectorizer is an important method for extracting features and vectoring data in a dictionary. When a discrete variable is represented in an unordered string value, it needs to be encoded by this method into numeric variables. Although the dictionary vectorizer can simply transfer the original data in string type or like fashion as a numeric dataset, it might be out of standard and far from practical applications since there will be some special data, in which the category is larger than other categories, and the categories in the original features have no size relationship. It can lead to a negative effect on model building and risk evaluation.

Considering the potential problem of encoding rules for food safety, one-hot encoder is introduced to continue completing the entire encoding. When a discrete variable is processed after the dictionary vectorizer into a feature vector without numerical order, the one-hot encoder can make further improvements on the encoding quality by distinguishing different categories of feature vectors. The one-hot encoder uses an *N*-bit register to encode *N* states of the feature vector, and each state has its register bit. Once the Euclidean distance between two states is the same, the corresponding register bit is valid at any time as 1, and others are 0. In particular, if there are original data in *m* × *l* dimensionality, which content *n*-type features in the *l* column, then the one-hot encoder uses the 0/1 binary coding to represent the feature vector with different types and changes the data dimensionality into the new format *m* × *n*. This encoding process does not alter any meaning of all fundamental features and ensures the Euclidean distances among different features with types are the same, so we could analyze the risk level based on some accepted unstructured data that occurred at a supply chain node.

#### 3.2.2. Value-Standardized Processing

For the same feature, the values of different samples may be far apart. Some abnormally small or big data may mislead the correct training of the model. Additionally, the data with very scattered distribution will also affect the training results since the classification results will be biased toward larger values. Value standardization of the database is a common requirement of most machine learning algorithms: if individual features do not look like standard normal distribution data (for example, Gaussian with 0 mean and unit variance), they may behave badly. At this point, we can normalize the values in the features, that is, convert to a normal distribution with a mean of 0 and a variance of 1. Thus, it is important to explore the data distribution of the features and consider whether it is necessary to standardize the data before training the model. In this paper, we complete the value standardization operation in the Python programming language by calculating the mean and standard deviation of the training set as well as the test dataset in the same transform. The standard score of sample *x* is calculated by removing the mean and scaling to unit variance as follows:

(1) $$\begin{array}{c} D=\frac{x-\mu_{j}}{s_{j}}, \end{array}$$

where *μ*_*j*_ is the mean of the training samples and *s*_*j*_ is the standard deviation. Relevant statistics for the samples in the training set are calculated, which can be centered and scaled independently of each feature. The mean and standard deviation are then stored to handle with subsequent data. The normalized variable values fluctuate around 0, with greater than 0 indicating above average; otherwise, it means below average. In a word, the correct and key data would be stored in the food system for the mining risk level and prewarning information after analyzing and processing unstructured abnormal data from the retrospective database.

### 3.3. The Deep-Stacking Network Method

Based on the preliminary analysis of IoT data, this study intends to find out inherent relationships among items in a huge database, which consists of key features with temporal and spatial distributions matching different hazard categories in grain products. However, it is still difficult to comprehensively analyze the risk grade of hazardous materials in the whole supply chain since the previous methods are not applicable to dispose a mass of heterogeneous data offered by multisource IoT sensors and instruments. Therefore, a novel deep-stacking network is proposed to accurately identify the risk level of main hazards and timely carry out risky prewarning of food products in the grain supply process. In order to improve the identification accuracy, this paper achieves the proposed method with three stages. Firstly, a multigranularity padded-scanning method is adopted to generate input vectors with adjustable dimensions in the first stage, and the *K*-fold cross-validation method is applied to expand the utilization of limited training data in the second stage. Finally, the deep-stacking network is consolidated to extract a list of food safety prewarning features, which speed up the mining process and improve identification performance in the present study.

#### 3.3.1. Multigranularity Padded-Scanning Method

Because the raw database is mostly unbalanced, multigranularity scanning is to utilize many sliding windows with different sizes and functions to filter the input dataset for forming new input vectors, which obtain the enhanced representation ability of high-dimensional input data. The basic scanning process is as follows: first, input a complete *M*-dimensional sample, and then perform sliding sampling through a filtering window of length *k* and step size 1 to obtain the subsample vector of *M*′-dimensional feature:

(2) $$\begin{array}{c} M^{\prime }=\left(M-k\right)+1. \end{array}$$

Each subsample is then used for training in partial or complete random forests, and a probability vector of length *P* is obtained in each forest. In this way, each forest will generate a characterization vector of length *M*′*∗P*. The results of different forest layers will be spliced together to obtain the output of this layer. The underlying multigranular scan will oversample all features except the first and last dimensional features, while the first and last dimensional features will be sampled less once. However, the first dimension and the last dimension feature may be very important in practical applications; that is, reducing the two-dimensional sampling relative to other dimensions may affect the final result of the classifier.

**Figure 2:**
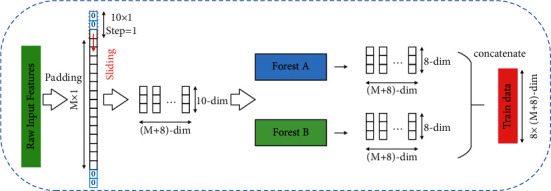
Multigranularity scanning process with padding optimization.

So, considering the limitation, this paper proposes a padding optimization to perform a zero-compensation operation on the edge of the *P*-dimensional sample. To this end, add an extra feature with value 0 and length *k* − 1 to both ends of the input *M*-dimensional sample before the original multigranularity scanning process, as shown in Figure 2. Then, the sampling window with the length of *k* and the step size *s* is subjected to sliding sampling so that the *k*-dimensional feature of the subsample vector is obtained as *M*′. The formula is determined by the following equation:

(3) $$\begin{array}{c} M^{\prime }=\frac{\left(M+2\left(k-1\right)-k\right)}{s}+1=\frac{\left(M+k-2\right)}{s}+1. \end{array}$$

According to the above multigranularity padded-scanning method, we could get the more effective input vectors with adjustable dimensions by scanning the feature with padding operation since most of the key information contained in the unbalanced samples is searched without losing too much raw data. Moreover, the subsequent methods can accurately determine whether raw data or feature vector should be focused on when an abnormality is detected, thereby availably avoiding the model overfitting in the risk analysis and evaluation process.

#### 3.3.2. *K*-Fold Cross-Validation Method

Using the results of the padded scanning as inputs, a simple hold-out validation is used; that is, *d* samples are randomly selected from all training data *D* as a training set, and the rest is used as a test set. However, in this case, the data were only used once and were not fully utilized. In order to make full use of the limited training database, we can use the *K*-fold cross-validation method to divide the training/test dataset into a larger number of subsets. After this operation, the model overfitting is further processed due to dataset bias and improper dataset partitioning. The training data are divided into *k* parts; one part is selected as the test part and the rest as the training part in each of *k* iterations. Then, the evaluation metric integrating different outputs of subsets is obtained as follows:

(4) $$\begin{array}{c} E=\frac{1}{k}\sum_{i-1}^{k}E_{i}, \end{array}$$

where *k* is the subset of the original sample *D* of equal size, *i*=1,2,3,…, *k* is the number of the *i*th subset, *E*_*i*_ is the evaluation results on each subset, and *E* is the overall evaluation result.

#### 3.3.3. Deep-Stacking Network Architecture

**Figure 3:**
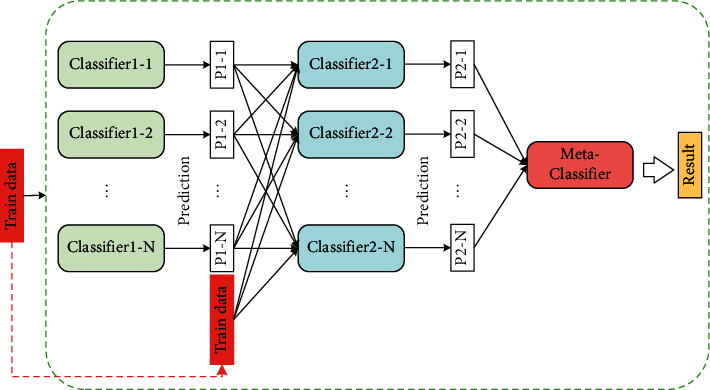
Structure diagram of the deep-stacking network method.

In order to promote the risk identifying capacity of the grain RITS, this paper introduces the deep-stacking network method inspired by the classic stacked learning strategy to mine the relationship among risk factors and hazards, meanwhile to assign the corresponding weights according to their different risk levels. The deep-stacking network algorithm first uses a large amount of data to train the primary learner and obtains the output of the first-layer learner through the primary learner. Next, a layer in the classic stacking algorithm is added, the original input data and the results obtained by the first layer are concatenated, and they are input into the second layer. Then, the second layer of the primary learner is trained to obtain the output of the second layer; finally, the results of the second layer of learners are concatenated, and the final result is obtained by the secondary learner. The structure diagram is shown in Figure 3. To unify various feature vectors of different learners into the same dimensional space, this paper adds the 1 × 1 convolutional layer followed by a batch normalization layer to outputting feature vectors of the metaclassifier; thereby, each probability distribution of component features is computed by the Gaussian expectation expression, which is described as follows:

(5) $$\begin{array}{c} P_{i}\left(\tilde{y|\theta_{i}}\right)=\frac{1}{\sqrt{2\pi \sigma_{i}}}\exp\left(-\frac{{\left(\tilde{y}-\mu_{i}\right)}^{2}}{2\sigma_{i}^{2}}\right), \end{array}$$

where *P*_*i*_ denotes the probability score for the *i*th component classifier. *θ*_*i*_=(*μ*_*i*_, *σ*_*i*_^2^) denotes the estimation parameters, which are composed of the mean vector *μ*_*i*_ and the covariance matrix *σ*_*i*_^2^, respectively. And $\tilde{y}=\left\{\tilde{y_{1}},\tilde{y_{2}},\ldots ,\tilde{y_{N}}\right\}$ is the output label vector corresponding to the input features, which should reflect the substantive features of their original data. Subsequently, a Gaussian mixing method is applied to count the final prediction result *P* of the risk level, which fuses the probability scores of different subclassifiers. The log-likelihood estimation of *P* is performed as the following expression:

(6) $$\begin{array}{c} P=\log\left(\sum_{i=1}^{n}P_{i}\right)=\prod_{i=1}^{n}\sum_{j=1}^{m}\gamma_{ji}\left[\log\left(\frac{1}{\sqrt{2\pi }}\right)-\log\;\sigma_{i}-\frac{1}{2\sigma_{i}^{2}}{\left(y_{j}-\mu_{i}\right)}^{2}\right], \end{array}$$

where *n* is the number of subclassifiers, *m* indicates the number of input data, and *γ*_*ji*_ denotes the hidden variable representing the mixture weight of the *i*th component classifier on the *j*th label vector *y*_*j*_∈$\tilde{y}$. With the constraint that the sum of *γ*_*ji*_ adds up to 1, the total probability distribution of the final result is normalized by maximizing the expected value of the log-likelihood function given in multiple iterations until the parameters reach convergence.

The overall algorithm steps are as follows: firstly, the training data are coded by the dictionary vectorizer and one-hot encoder, and the value is standardized according to different hazard categories in the grain; secondly, the multigranularity scanning method is improved, and all features are extracted equally by adding padding operation; meanwhile, the *K*-fold cross-validation is used to divide the raw database into *k* equal-sized subsets to train multiple different learners of the first layer; next, the output result of the layer is concatenated with the original input as an input of the next layer, and a plurality of different learners of the second layer are trained; finally, in the case of multiple trainings, the accuracy is no longer improved, the training results of the last layer of learners are merged to obtain the final results, and the model is subsequently applied for testing new data. The overall pseudo-code of the deep-stacking network framework for the decision-making process is shown in Algorithm 1.

## 4. Experimental Results

### 4.1. Comparative Results

In order to illustrate the applicability and effectiveness of the improved RITS with the support of the deep-stacking network method, a case study has been conducted in the grain industrial chains. In order to prove the scientific and the effectiveness of this research, the grain datasets describing 34 and 170 instances were selected from the retrospective database stored in the cloud platform of the RITS to train and test the risk evaluation model. The original sample datasets contain 33 attributive indicators and the given risk level, of which 14 features are coefficient terms recorded as string-type data, and the others are recorded as numerical features. The category distribution of all instances according to eight risk levels is as follows: level I (15566), level II (3752), level III (1288), level IV (2117), level V (2575), level VI (1726), level VII (1386), and level VIII (5760).

The nature of this dataset allows for multiple risk levels to be present in each instance. Therefore, all raw data were partitioned into 90% and 10% splits of training and testing subsets for *K*-fold cross-validation with *k* = 5. Stratified random partitioning was performed to ensure the even distribution of the classes within each level subset, which affects the feasibility of training and testing the model. The 90% random split constitutes the training subset, while the remaining 10% were used as the validation subset to monitor the training process and minimize overfitting. The random splits for each fold were controlled by a random seed such that the individual split could be reproduced as required.

When the best choice of the training process and parameters is achieved, the trained model was used to predict the unknown risk level of the 10% testing datasets. The prediction result of remaining data was then compared with the given risk level. To objectively evaluate the results of different algorithms, four performance indicators including accuracy (ACC), precision (PRE), recall (REC), and *F*1-score (*F*1) were used to compare the prediction results of each model. Among them, the ACC is the ratio of the number of correctly marked items to the total number of observations. And the PRE is the number of true positives divided by the total number of items marked as belonging to that category.

Meanwhile, the REC is defined as the number of true positives divided by the total number of items belonging to that category, also known as sensitivity. Through the above three indicators, the number of results in the confusion matrix can be converted into a ratio between 0 and 1, which can be counted on the basis of true positive (TP), true negative (TN), false positive (FP), and false negative (FN). Moreover, this paper employs another high-level indicator *F*1-score, which is the harmonic mean of the ACC and REC with better comprehensive evaluation for multicategory identification problems. The detailed definitions of indicators are as follows:

(7) $$\begin{array}{c} \begin{array}{c} \text{ACC}=\frac{\text{TP}+\text{TN}}{\text{TP}+\text{TN}+\text{FP}+\text{FN}} \end{array},\\ \begin{array}{c} \text{PRE}=\frac{\text{TP}}{\text{TP}+\text{FP}}, \end{array}\\ \begin{array}{c} \text{REC}=\frac{\text{TP}}{\text{TP}+\text{FN}}, \end{array}\\ \begin{array}{c} F_{1}=2\times \frac{\text{PRC}\times \text{REC}}{\text{PRE}+\text{REC}} \end{array}. \end{array}$$

To verify that the proposed method could be used in the grain supply chain for recognizing risk levels, we establish some experiments to compare the performance of the deep-stacking network with that of other methods. Some classical machine learning methods including logistic regression (LR), *K*-nearest neighbour (KNN), support vector machine (SVM), extra trees (ET), gradient boosting (GB), random forest (RF), and decision tree (DT) and five deep learning models including AlexNet, VGG, GoogLeNet (GLNet), ResNet, and deep forests (DeepF) were selected in this section to illustrate the identification challenge in the grain supply chain, which has achieved remarkable success in other fields. All models are trained and tested on a cloud server platform with Ubuntu 16.04 system. And codes are based on the open-source framework, TensorFlow 2.3 with Python API, and run on a dual-core Intel Core i7-6800@3.6 GHz processor with four NVIDIA Tesla p40 GPUs, which have 96 G computing caches and 256 G memory. Before inputting into the model learning process, all original data were encoded and standardized to improve the robustness of the model.

In order to illustrate the effect of the multigranularity scanning and cross-validation operation on the accuracy, we conducted separate experiments of each model in Architecture-1 (without padded-scanning and cross-validation operations) and Architecture-2 (with corresponding operations). The identification accuracy results of each model are summarized in Table 3. It is demonstrated that the identification accuracy of the risk level during the grain product supply chain has been notably improved by the proposed DSN model, which had the best performance compared to other models.

As shown in Table 3, compared with other state-of-the-art models, the experiments showed that our proposed model has achieved comparable or better results for risk level identification on the accuracy. In the model list from top to bottom, the methods are separated into three groups, which are (1) machine learning models, (2) deep learning networks, and (3) proposed DSN. We first observe that the accuracy results of some machine learning methods are generally better than some deep learning models on the food safety dataset. For instance, the AlexNet and VGG are the classical conventional neural networks for various classification and detection tasks based on images or videos in other domains. However, they have achieved lower recognition rates than a few shallow-structured models including GB, RF, and DT, which illustrates the challenge and difficulty of automatic risk level recognition in complex food supply chains. With the complexity of the network structure optimized with various modeling tricks, deep learning networks began to show better accuracy results over traditional methods.

For example, GoogLeNet, ResNet, and deep forests design the complex nonlinear multilayer architecture to exploit discriminative high-dimensional features, achieving accuracy values up to 85.18%, 87.26%, and 90.73%, respectively. Meanwhile, with the less parametric architecture, the DSN method could produce the preferable accuracy by 94.88% even without the aid of padded-scanning and cross-validation operations, which have obviously improved the result by 9.7%, 7.62%, and 4.15%, respectively, in comparison with the above three deep learning models. It illustrates the advantage of a deep-stacking strategy in improving the efficiency and characterization capability of food risk prewarning. Moreover, with the extra support of multigranularity padded-scanning and *K*-fold cross-validation operation, all models achieved better average accuracies than original baselines as listed in the Architecture-2 column, which directly proves the necessity of related operations.

Moreover, this proposed approach does not add much computational burden to the recognition system in terms of model parameters, which achieves comparable and even outperforming results compared with other methods. In order to better evaluate the computing cost of each model, we select the model parameters as the metric, which indicates how much computer memory is occupied when the model is trained. In this paper, we count up the memory scales of conserved model weights to record the value of model parameters. As shown in Table 3, the comparison performance between our proposed DSN and other models suggested evident accuracy and computational advantages for risk level identification. For instance, compared with deep learning models including AlexNet, VGG, GoogLeNet, ResNet, and deep forests, the model parameters of Architecture-1 and Architecture-2 are 202.53 megabytes and 211.26 megabytes, respectively, which have been obviously improved at least by 16.43 megabytes and 12.28 megabytes. Although the other machine learning models with shallow structures obtain the smaller model parameters over most of the deep learning models and our DSN method, however, the overall performance of the DSN in both accuracy and model parameters is evidently larger, making it easy to build and embed in RITS applications.

**Figure 4:**
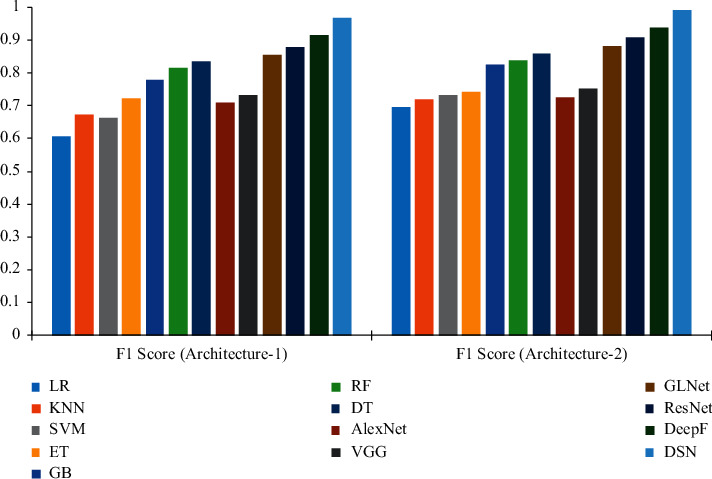
Structure diagram of the deep-stacking network method.

Besides, our DSN method achieves the best result with 97.62% accuracy in automatically and accurately identifying grain risk levels, which clearly performs over comparative methods. Similarly, the DSN method enjoys consistent better performance on the *F*1 indicator outperforming existing methods under the same baselines and has achieved large-margin promotion in terms of *F*1-score up to 0.967 under Architecture-1 and 0.991 under Architecture-2. This clearly indicates that feature learning and risk forecasting capability can still be improved even though the food data are very tricky for other methods. Therefore, our method is more suitable for risk level recognition in the food supply chains. The *F*1-score results of different models in two architectures are shown in Figure 4.

### 4.2. Predicting Evaluation of Each Risk Level

More comparisons of prediction results in terms of different risk levels are provided in Table 4, which can further illustrate the feasibility and effectiveness of the proposed DSN model. To properly assess the algorithm capabilities, new 7886 data from the grain database were used to calculate the PRE, REC, and *F*1 results for each risk category. When the PRE of a risk level is close to 1.00, it means that 100% of the security level instances in the test set are assigned to this correct level. And REC equal to 1.00 means that each item in the certain level belongs to the correct level, but does not indicate how many other items are incorrectly marked. Additionally, the range of the *F*1-score is from 0 to 1, with 1 representing the best output and 0 representing the worst output of the model. Therefore, the higher the value of three indicators, the stronger the model's ability for identifying and predicting.

As shown in Table 4, the proposed DSN method obtains the high average PRE, REC, and *F*1 values up to 0.99 for each risk level. The comparison of different risk levels revealed that level IV acquires the lowest values due to the small value divide interval between safety and risky concepts, which is still important but hard to accurately identify and assess. The results of the DSN indicate that the deep-stacking strategy has a great influence on the learning of features while performing network convergence, which further proves that the DSN model is more suitable for the recognition task of risk level recognition in grain supply chains because its stability is higher than the other methods.

**Figure 5:**
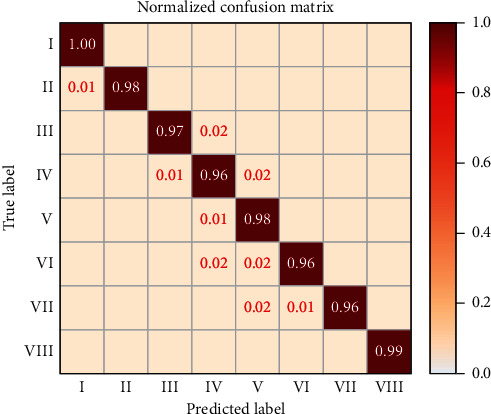
Normalized confusion matrix.

Moreover, the confusion matrix is used to measure the prediction performance of the DSN model for each risk level, which is the most basic, intuitive, and simplest method to assess the right and wrong distribution of the model outcome. The normalized confusion matrix of the DSN is shown in Figure 5, which further reflects the high correctness of predictability and low confusion of error division for grain risk prewarning.

### 4.3. Hyperparameter Influence on the Model Performance

Although many deep learning models have shown their efficacy for application in various fields, they may not be suitable for different kinds of datasets or problems. The main drawback restricting the expanded application of deep learning methods is that their performance will be seriously influenced by hyperparameters based on experiential knowledge or many attempts to select parameters for better results. This process may spend a lot of computing resources and time, and the hyperparameters obtained may not be optimal, resulting in the instability and limited accuracy of models. Therefore, making an effective experiment to discuss the optimal hyperparameters is an essential step to improving the model's performance. To solve the above issue, we investigate the three main hyperparameters including epochs, batch size, and *K* to analyze the influence. The relation between several hyperparameters and the model's accuracy is shown as follows.

**Figure 6:**
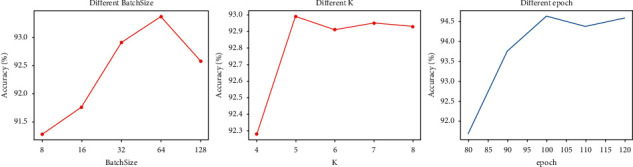
The influence of different hyperparameters on the model performance.

As shown in Figure 6, in the initial stage, as the batch size hyperparameter becomes larger, the model performance continues to rise, but after 64, the accuracy shows a downward trend. Therefore, the best selection of batch size should be 64 when other conditions are the same. Similarly, with the increase of the *K* hyperparameter, the accuracy of the model increases rapidly. The difference is that the accuracy tends to be stable and even fluctuates when the value reaches 5. This means that the best value of *K* is 5, and there is no need to choose a higher value. It will not only increase the amount of calculation but also affect the stability of the model. Finally, it can be seen from the relationship diagram of the epoch selection that the performance is achieved when the epoch hyperparameter is 100 when other conditions are set the same. Through the above discussion, the hyperparameters of batch size, *K*, and epoch in this article are, respectively, set as 64, 5, and 100, in order to ensure the optimal performance of the proposed method.

### 4.4. Case Analyses and Discussion

In the rest of this paper, we study the risk level distribution of different hazards and analyze the relative effect on each supply link in order to offer vital prewarning rules for the whole food system as well as provide a detailed explanation related to the superiority of the DSN model for food safety assurance. Through the above experiments, we can know that the risk situation of the hazardous materials is different in each supply chain link, but the risk level of various hazards may gradually accumulate and spread along with supply chain processes. When these risk levels and related features were input into the RITS for a period, we found the results are encouraging to make operators find the food safety risk immediately and enhance the food quality assurance during the whole supply chain.

Moreover, with fully utilizing the IoT and cloud analysis technology, the prewarning rules could be mined by the proposed deep-stacking method from the massive unstructured data stored in the cloud platform. All generated prewarning information would be fed back into the food system and history database to allow a continuous and interactive process to achieve superior solutions and traceable schemes for the future possible problems in supply chain management. When more effective information and communication are shared among supply chain partners with the support of an IoT-based food system, the hidden food safety information would be visualized. The prewarning information created in the supply chain along with the product transactions would be analyzed by operators in different food industries and companies, and all such results are extracted and displayed in a visualization way, through which the decision-making gap between supervisors and consumers can be more clearly understood and offset.

**Figure 7:**
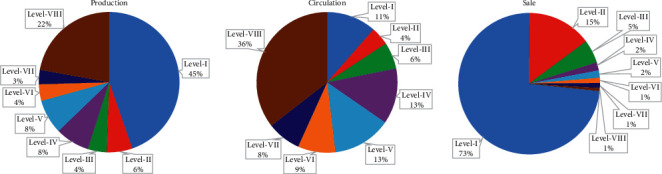
Risk level distribution in grain supply links.

For example, we could observe the distribution discrepancy of each risk level among grain supply chains as in Figure 7. It can be seen that the products under level I and level II occupy the majority of all grain products, where the overall quality of the supply chain is very safe. Nevertheless, in the production and circulation links, level VIII and above products account for a large proportion, indicating that these products are highly prone to risks due to environmental changes and spatiotemporal accumulation in a wide range. Therefore, food regulatory agencies need to strengthen the supervision of the front-end links when conducting random food safety inspections to protect the health of consumers. Meanwhile, enterprise managers should pay much attention to the quality and safety of grain in production and circulation links and should add more effective monitoring and prewarning technologies to avoid the food safety incidents to a significant extent.

**Figure 8:**
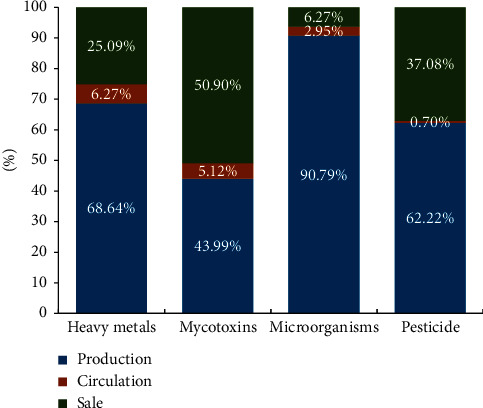
Risk distribution proportion of dominating contaminants in each supply link.

In addition, the food risk identification and prewarning system with the DSN method will help to analyze the relationship among various hazards, minimize the production of unsafe quality products, and reduce the economic losses by avoiding product recall activities or additional destruction operations. For instance, with the prewarning analysis of the potential relationship between the hazard category and supply links, the prevention and control rules would be more scientific, while the operators always determine the corresponding operations on the basis of personal experience before. As shown in Figure 8, the ratio graph of unqualified samples in the production, circulation, and sales links is shown to illustrate the link distribution of four dominating contaminants.

We could find that the production and sales links suffered a greater threat of various pollutants. Nearly 70% of the heavy metal hazards are detected in the production link originating from the metal residue of polluted soil and irrigation water. Heavy metals exceeding the standard are also frequently detected in other links, which means that heavy metals easily continue to migrate and spread in the grain supply chain. The excessive situation of pesticides has a similar proportional distribution except for the sale link, indicating that there are lots of excessive additives and unqualified operations in the subsequent process of sales and consumption, which need to be focused on and prevented in grain supply chain management. The typical issue is that the illegal addition of benzoyl peroxide (BP) which has been confirmed to damage the human body and forbidden in the Chinese national standard has increased in wheat flour and related products.

Meanwhile, most microorganisms are concentrated in the early production links with 90.79%, which reveals that the grain safety risks of microorganisms are mainly caused by the production industries and processing enterprises since most microorganisms are sensitive to many environmental factors of the global grain supply chain including transit time, temperature, humidity, conveyance, and product type. In contrast, the mycotoxins are affected by more complicated factors in all links of the grain supply chain, where there are various intrusion sources. The distribution ratio also reveals that mycotoxins can exist for a long time with biodegradability degradation resistance, which are easy to migrate and enrich in continuous links of the grain supply chain. Thereby, with the support of the risk identifying and prewarning system, the performance improvement of risk identification and prewarning enhances the safety and quality level of food supply chains, which even promotes the management efficiency of the food crisis and prevents the interruption of sustainable food supply and the food recall cost, and waste would also be reduced.

**Figure 9:**
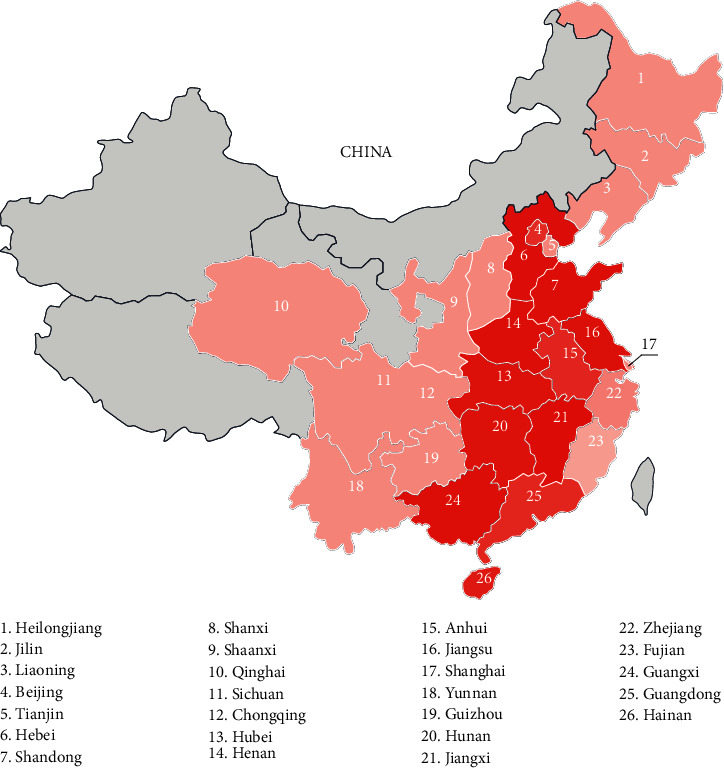
Geographical distribution of unqualified products, where the darker the color of provinces, the more the unqualified samples.

For example, with the geographical analysis of unqualified grain products in different provinces as shown in Figure 9, customers will obtain an intuitive and comprehensive perception of the prewarning rules between unqualified production and geographical distribution. It can be seen from Figure 1 that there are more cases of unqualified grain in the southeastern coastal areas, while there are fewer cases of grain unqualified in the western and northeastern regions, which may depend on the distribution of grain, the population density of different provinces, and the level of consumption. So, consumers gain better confidence, security, and satisfaction with access to safe, high-quality production. Besides, the regional differences reveal the urgent requirement for the overall supervision of grain products across the whole supply chain, which calls policymakers to provide infrastructure development and financial assistance to enterprises. There is also an urgent need for the implementation of the intelligent food system with the help of IoT technology and cloud analysis, which reflects the society's emphasis on the security and sustainability of grain supply chains.

All in all, the developed DSN method has provided an effective approach for the food risk identifying and prewarning system to strengthen the quality, security, and sustainability of food supply chains. With the support of IoT, cloud analysis, artificial intelligence, and other technologies, it becomes easier to allow various food enterprises to effectively monitor, identify, and predict risk levels in real time. Accurately identifying and sharing of risky factors can reduce recall costs and unnecessary waste while promoting the consumers' satisfaction and security for high-quality food productions. The system also provides more prewarning information for policymakers to develop targeted regulatory policies and provide low-risk financial assistance with a comprehensive decision-making perspective to ensure the grain supply chains' quality sustainability. Experimental results and analyzing discussion of the practical cases validate the feasibility of adopting the proposed DSN model embedded into the IoT-based RITS, which would avoid food safety incidents to a significant extent.

## 5. Conclusion

Overall, this study has proposed a new deep-stacking network for the IoT-based risk identification and traceability system to identify risk levels and mine traceability rules in food supply chains. With the support of IoT technology and cloud analysis platform, the system would monitor massive multisource biosignals about food supply safety through various sensors and instruments in real time. Then, a deep-stacking network optimized with data mining is proposed to enhance the efficiency and performance of the RITS for guaranteeing food supply chain security sustainability. The verification experiments with practical food safety datasets show that the proposed method has the best performance in terms of prediction accuracy and stability compared to other models, which takes various indicators reflecting identification accuracy, response speed, and computing consumption of food systems into account. Thereby, this work indeed makes a significant contribution to promoting the security and sustainability of food supply chains, as well as mining important decision support rules to help regulatory authorities, enterprises, and consumers.

The proposed methods in this paper can combine other identification schemes for studying new modeling and prediction of dynamical systems [40–42] and can be applied to other fields [43–46] such as signal modeling, tracking, and control systems. On the contrary, our network has high requirements for data and does not perform well for unstructured data. At the same time, this method has not been verified in other scenarios. Therefore, we would like to apply advanced deep learning methods to adjust hyperparameter searching and model training to improve the overall reliability and efficiency of the food safety system even further.

## Figures and Tables

**Algorithm 1:**
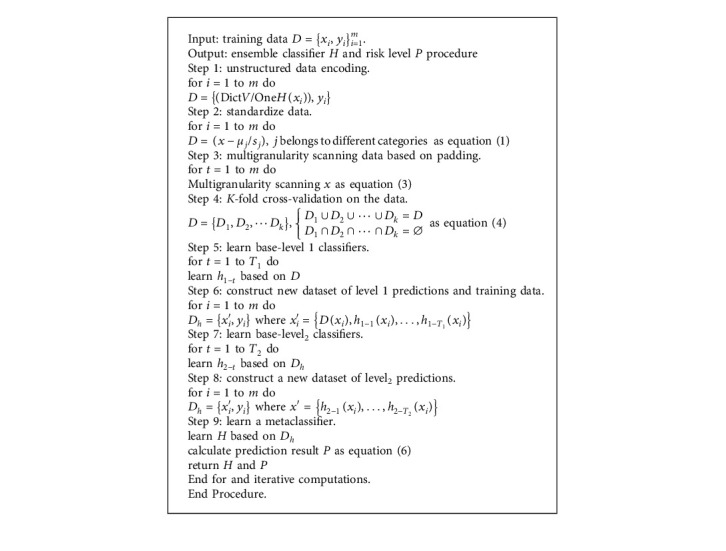
Deep-stacking network flowchart.

**Table 1:** Attributive categories of the grain database.

Attributive name	Value
Province	Anhui | Beijing | Chongqing | Fujian | Guangdong | Guangxi | Guizhou | Hainan | Hebei | Heilongjiang | Henan | Hubei | Hunan | Jiangsu | Jiangxi | Jilin | Liaoning | Qinghai | Shaanxi | Shandong | Shanghai | Shanxi | Sichuan | Tianjin | Yunnan | Zhejiang
Grain type	Rice | wheat | corn | roughage| rice-processed products | wheat-processed products | else
Link	Production | circulation | sale
Production area	City | village
Sampling sites	Planting bases | warehouse | workshop| transportation facility | farmer markets | supermarkets |restaurants | hotel| laboratories|
Hazard type	AFB1| OTA| ZON| DON| T2| fumonisin| Al | As| Cd| Cr| Hg| Pb | COLI | *Salmonella*| MRSA| tebuconazole| benzopyrene (BaP) | malathion|
Risk item	Heavy metals| microorganisms| mycotoxins| pesticide residues|
Content and unit	mg/kg | *μ*g/kg | CFU/g | MPN/g
Temperature	−10°C–40°C
Humidity	0%–100%
Light exposure	0 Lux–1500 Lux
Oxygen	17%–25%
Carbon dioxide	0 ppm–1200 ppm
Weight	0 kg–100 kg
Expiration date	3–24 months
Production data	2015.01–2019.07
Risk level	I | II | III | IV | V | VI | VII | VIII

**Table 2:** Extended attributes of the grain database.

Dangerousness	Sociality	Economic	Regulatory
Health guidance value Median lethal dose (LD50) Acceptable daily intake (ADI) Short-term dietary intake (IESTI) Carcinogenicity Toxicity	Social attention Security Event frequency Hot search index (Google and Baidu)	Annual total output of the province Annual total planting area of the province Grain production price Grain consumption price	Regulatory accessibilityStandard quantity

**Table 3:** Identification result of the risk level in comparative methods.

Models	Accuracy (%)		Model parameters (megabyte)	
	Architecture-1	Architecture-2	Architecture-1	Architecture-2
Logistic regression [19]	59.21	69.35	41.31	43.25
*K*-nearest neighbour [35]	66.32	69.01	50.65	61.36
Support vector machine [20]	66.70	72.60	47.33	58.97
Extra trees [18]	71.88	72.83	63.57	70.65
Gradient boosting [22]	78.94	81.29	61.37	66.91
Random forest [26]	80.51	83.93	70.18	81.32
Decision tree [17]	83.97	85.45	72.34	79.25
AlexNet [26]	70.62	71.32	218.96	223.54
VGG [28]	72.88	73.94	320.78	365.12
GoogLeNet [27]	85.18	87.98	425.89	478.23
ResNet [29]	87.26	89.68	507.34	528.67
Deep forests [32]	90.73	92.47	385.32	419.11
Proposed DSN	94.88	97.62	202.53	211.26

**Table 4:** Recall, precision, and *F*1-score of each risk level based on the proposed model.

Level	PRE	REC	*F*1-score
I	1.00	1.00	1.00
II	0.98	0.98	0.98
III	0.97	0.97	0.97
IV	0.95	0.96	0.96
V	0.95	0.98	0.97
VI	0.97	0.96	0.97
VII	097	0.96	0.97
VIII	1.00	0.99	0.99
Total average	0.99	0.99	0.99

## Data Availability

The food safety data used to support the findings of this study are available from the corresponding authors upon request.
